# Analysis of the *TP53* Deleterious Single Nucleotide Polymorphisms Impact on Estrogen Receptor Alpha-p53 Interaction: A Machine Learning Approach

**DOI:** 10.3390/ijms20122962

**Published:** 2019-06-18

**Authors:** Kumaraswamy Naidu Chitrala, Mitzi Nagarkatti, Prakash Nagarkatti, Suneetha Yeguvapalli

**Affiliations:** 1 Department of Zoology, Sri Venkateswara University, Tirupati 517502, India; cksnaidu@gmail.com; 2 Department of Pathology, Microbiology and Immunology, University of South Carolina School of Medicine, Columbia, SC 29208, USA; Mitzi.Nagarkatti@uscmed.sc.edu (M.N.); prakash@mailbox.sc.edu (P.N.)

**Keywords:** breast cancer, *TP53*, ERα, single nucleotide polymorphism, genetic factors, machine learning

## Abstract

Breast cancer is a leading cancer type and one of the major health issues faced by women around the world. Some of its major risk factors include body mass index, hormone replacement therapy, family history and germline mutations. Of these risk factors, estrogen levels play a crucial role. Among the estrogen receptors, estrogen receptor alpha (ERα) is known to interact with tumor suppressor protein p53 directly thereby repressing its function. Previously, we have studied the impact of deleterious breast cancer-associated non-synonymous single nucleotide polymorphisms (nsnps) rs11540654 (R110P), rs17849781 (P278A) and rs28934874 (P151T) in *TP53* gene on the p53 DNA-binding core domain. In the present study, we aimed to analyze the impact of these mutations on p53–ERα interaction. To this end, we, have modelled the full-length structure of human p53 and validated its quality using PROCHECK and subjected it to energy minimization using NOMAD-Ref web server. Three-dimensional structure of ERα activation function-2 (AF-2) domain was downloaded from the protein data bank. Interactions between the modelled native and mutant (R110P, P278A, P151T) p53 with ERα was studied using ZDOCK. Machine learning predictions on the interactions were performed using Weka software. Results from the protein–protein docking showed that the atoms, residues and solvent accessibility surface area (SASA) at the interface was increased in both p53 and ERα for R110P mutation compared to the native complexes indicating that the mutation R110P has more impact on the p53–ERα interaction compared to the other two mutants. Mutations P151T and P278A, on the other hand, showed a large deviation from the native p53-ERα complex in atoms and residues at the surface. Further, results from artificial neural network analysis showed that these structural features are important for predicting the impact of these three mutations on p53–ERα interaction. Overall, these three mutations showed a large deviation in total SASA in both p53 and ERα. In conclusion, results from our study will be crucial in making the decisions for hormone-based therapies against breast cancer.

## 1. Introduction

Breast cancer is one of the leading causes of cancer deaths faced by women around the world today and is a major health issue faced in the western part of the world. The burden of breast cancer is not evenly distributed showing a large variation in the incidence, mortality and survival between different countries and regions and within specific regions [1]. Recent reports showed an increase in the incidence of breast cancer in low and middle-income countries with an approximately 45% of new cases being diagnosed each year and more than 55% of deaths being occurred in low and middle-income countries [2,3,4]. In Asian countries like India, its prevalence is increasing slowly with an increasing incidence rising from 0.5–2% across all regions of India and in all age groups with an average age group found to be 35 years of age [5].

Alcohol intake, body mass index, hormone replacement therapy with estrogen and progesterone, radiation exposure, early menarche, late menopause, age at first childbirth, current age, past history of breast cancer, family history and germline mutations are some of the non-genetic factors that confer risk to breast cancer [6]. Among these risk factors, prolonged exposure to sex steroid estrogen (early menarche, late menopause, or postmenopausal hormone replacement therapy) is associated with a high breast cancer risk [7]. Estrogen is a female hormone secreted mainly by the ovaries to proliferate the endometrium as a part of the menstrual cycle. Functions of estrogen include the promotion of subcutaneous fat accumulation, mammary gland proliferation, water and sodium retention and calcium deposition [8]. Before menopause, it stimulates the vaginal epithelial cells to produce large amounts of glycogen and after menopause, its decreased levels cause lowering of glycogen content [9]. Estrogen ablation or anti-estrogen strategy is an effective means of prevention or treatment of breast cancer, especially in estrogen receptors (ERs)-dependent breast cancer.

Estrogen exerts its effects through two types of specific receptors i.e., estrogen receptor alpha (ERα) and estrogen receptor beta (ERβ). Both ERα and ERβ are ligand-dependent transcription factors mediating the biological effects of estrogens and antiestrogens [10]. Both ERα and ERβ are the members of a superfamily of genes that contain nuclear receptors for diverse hydrophobic ligands such as steroid hormones (estrogens, progestins, glucocorticoids, mineralocorticoids), retinoic acids (vitamin A), vitamin D, prostaglandins, and thyroid hormones [11]. Among ERα and ERβ, the clinical role of ERα has only been established and it plays a prominent role in the etiology of breast cancer and with respect to the prognosis of breast cancer, it has been extensively studied.

An in vitro study on ERα showed that, though ERα associate with the chemoresistance of breast cancer, ERα itself does not mediate this resistance process [12]. It prevents p53-dependent apoptosis in breast cancer [13] and is known to interact with the other receptors like Retinoic acid receptor α which is a known estrogen target gene in breast cancer cells [14]. ERα interacts with tumor suppressor protein p53 directly and represses its function thereby promoting the proliferation of breast cancer cells [15]. However, it is known that the physical interaction between p53 and ERα interferes with each other’s activities to regulate gene expression [16]. Histone deacetylase 1 binds to p53 and down-regulates its transactivation function, which is largely dependent on the 30 C-terminal residues of p53. The same region of p53 is known to be the domain that interacts with ERα [17]. Binding of ERα with p53 leads to the repressing of its functions in breast cancer cells, indicating that inhibition of p53 functions by ERα contributes to the inactivation of p53 [18].

**Figure 1:**
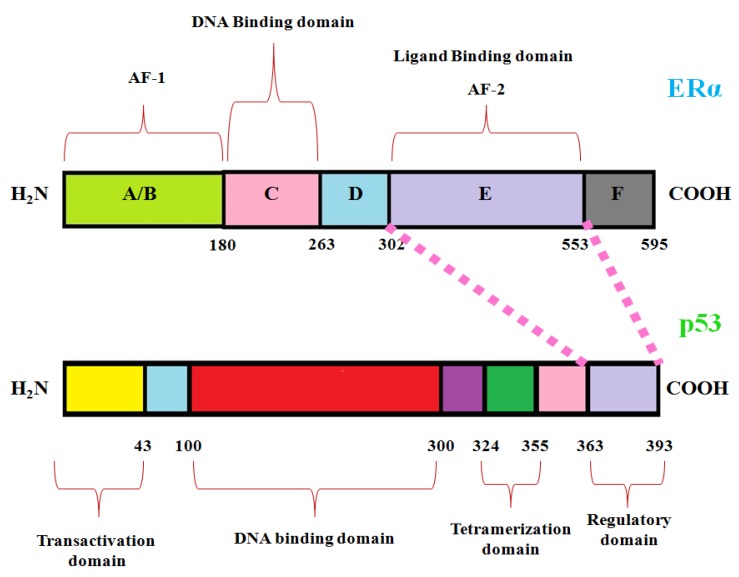
Schematic representation of interaction between ERα and p53. Region important for ERα–p53 interaction is shown in pink colour dotted lines. ERα domain regions were shown in the top and p53 domain regions were shown at the bottom. AF1 refers to constitutive transcription activation 1 domain and AF2 refers to the transcription-activating function 2.

The ERα consists of 595 amino acids with a molecular weight of 66 kDa separated into six different functional domains (A–F) [19]. Among them, the activation function-2 (AF-2) domain of ERα and the C-terminal regulatory domain of p53 are necessary for the interaction [15]. The respective interaction of ERα with p53 is shown in Figure 1 given below. Here, in the present study we analyzed the impact of p53 DNA binding domain deleterious breast cancer-associated nSNPs predicted in our previous study [20] on the p53–ERα interaction.

## 2. Results

### 2.1. Modeling the p53 Full-Length Protein Structure

**Figure 2:**
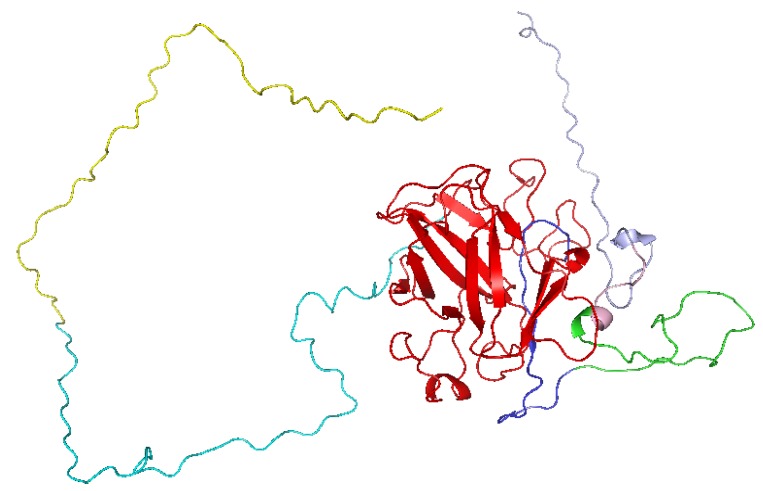
Three-dimensional structure of full length p53 structure. Transactivation domain is represented in yellow, DNA binding domain is shown in red, tetramerization domain is shown in green and regulatory domain is shown in light blue color.

A BLAST search for respective domains of p53 showed several hits. Among them, the one with the highest similarity was considered for modelling. Respective templates used for the domains in p53 were shown in Table 1 given below. Among several 3D models generated using homology modelling, the best model was selected after a series of refining and minimization. The three-dimensional structure of the generated model is shown in Figure 2 given below. Ramachandran plot drawn through the PROCHECK program validated the model with 90.1% of the total residues in the most favored regions and 9.9% in the additional allowed regions. None of the residues were located in the disallowed region confirming that the protein backbone dihedral angles phi (Φ) and psi (Ψ) occupied reasonably accurate positions in the selected 3D model.

### 2.2. Impact of p53 Mutants on p53–ERα Interaction

**Figure 3:**
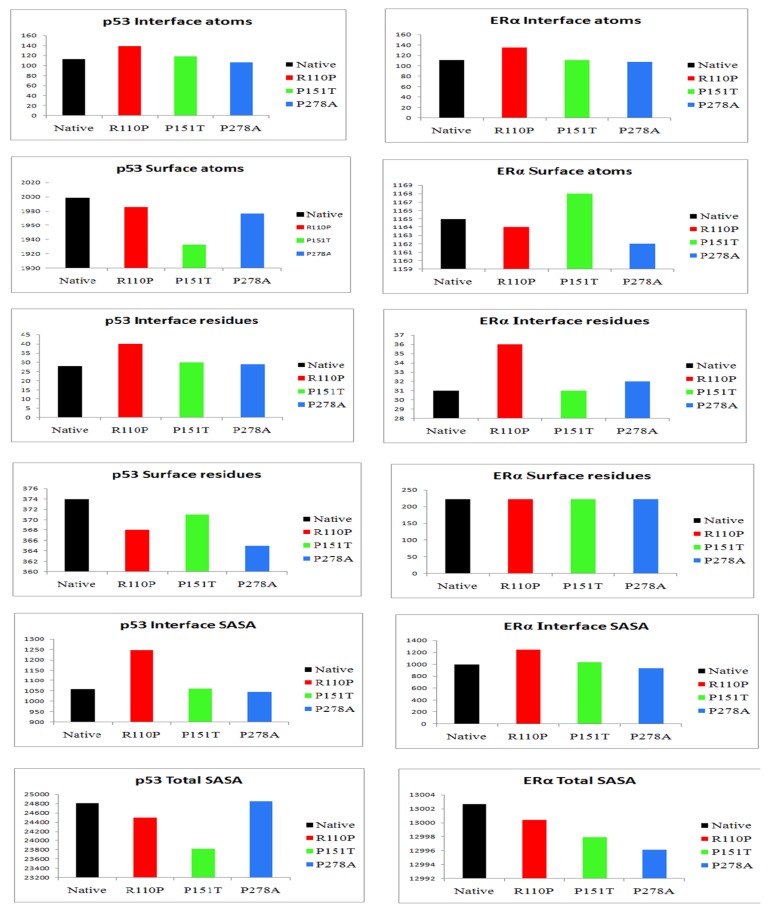
Surface, interface and solvent accessible surface analysis of the impact of three mutants R110P, P151T and P278A on the p53–ERα interaction. Black: native or WT, Red: R110P, Green: P151T, Blue: P278A.

**Figure 4:**
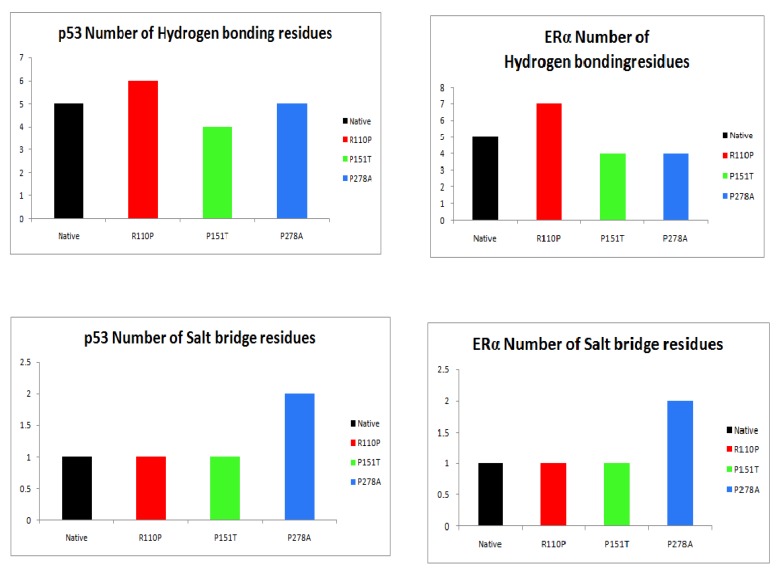
Analysis of the number of hydrogen bonding and salt bridge residues in p53-ERα complexes. Black: native or WT, Red: R110P, Green: P151T, Blue: P278A.

**Figure 5:**
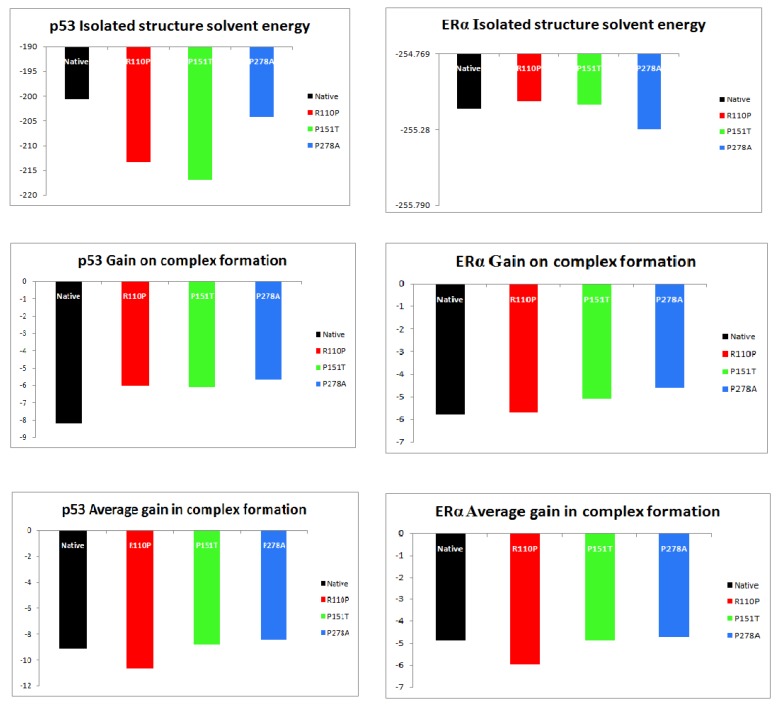
Analysis of the isolated solvent energy and gain in complex formation in p53-ERα complexes. Black: native or WT, Red: R110P, Green: P151T, Blue: P278A.

Protein–protein docking between p53 and ERα from ZDOCK analysis resulted in ten complexes each for native, R110P, P151T and P278A. The average of the properties of these complexes showed that interface atoms, interface residues and interface SASA was increased in both p53 and ERα for R110P compared to the native complexes indicating that R110P have more impact on the p53–ERα interaction on the interface compared to the other mutants. P151T and P278A, on the other hand, showed a large deviation from the native p53-ERα complex in the surface atoms and surface residues. Overall, all three mutants showed a large deviation in the total solvent accessible surface area in both p53 and ERα (Figure 3). Analysis of the number of hydrogen bonding residues and the number of salt bridges showed that an increase in the number of hydrogen bonding residues was shown by R110P compared to native complex whereas an increase in number salt bridge forming residues were showed by P278A (Figure 4). Analysis of structure solvent energy and the average gain in the complex formation properties showed all the three mutants deviating from the native indicating that all these three mutants have an impact on the p53–ERα interaction (Figure 5). Overall, these results indicate that all these three mutants have an impact on the p53–ERα interaction.

### 2.3. Artificial Neural Network Analysis

**Figure 6:**
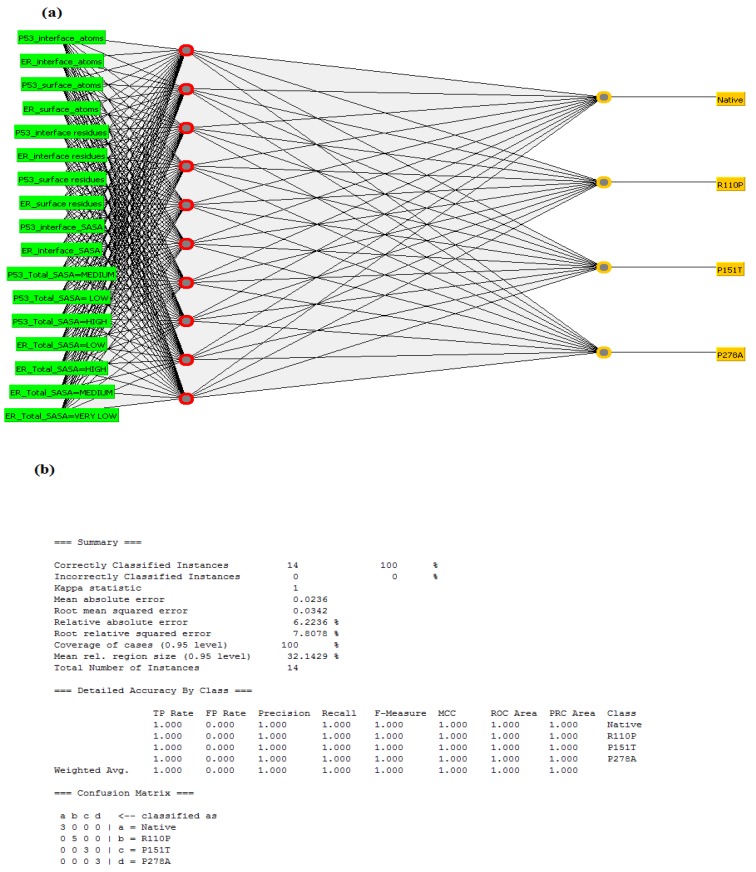
Neural network architecture for identification of the impact of mutants on p53–ERα interaction (**a**) Represents the neural network architecture for identification of the impact of mutants on p53–ERα interaction. The input layer is the pre-analysis dataset given in the Table 3 and the output layer has four output nodes with native at the left and remaining three nodes for the three mutants R110P, P151T, P278A (**b**) Represents the summary information of the neural network.

Results from the protein–protein docking analysis showed that all three mutants are known to show an impact on the interaction between p53 and ERα. We used this dataset for building an artificial neural network. These neural networks are nothing but, simple elements operating in parallel. The network function is determined largely by the connections between elements. We used a multilayer perceptron function for building the neural network. Multilayer perceptron involves building the class prediction function using backpropagation for minimizing the errors during learning by adjusting the weights of the connections between the network’s nodes [21]. The general architecture of a multilayer perceptron involves an input layer, a hidden layer, and an output layer. The respective attributes and dataset pre-analysis used for building the neural network for the p53–ERα interaction is given in Table 2 and Table 3 given below. Results from the neural network analysis showed that pre-analysis datasets given in the Table 3 showed 100 percent correct instances compared to the including all the attributes that are given in Table 2 indicating that these structural features are important for predicting the impact of these three mutants on the p53–ERα interaction. The respective artificial neural network architecture and its summary information are given in Figure 6a,b given below.

## 3. Materials and Methods

### 3.1. Full-Length Structure of p53

To investigate the mechanism of the impact of deleterious mutants R110P, P151T and P278A on the interaction with estrogen receptor alpha, we used a protein–protein interaction study. The size of the p53 tetramer is about 43 kDa for the monomer and less than 200 kDa for the tetramer, making it one of the smallest proteins ever studied by electron microscopy single particle reconstruction. Two different EM studies obtained different results. To the best of our knowledge the complete structure of the p53 protein is not available [22]. With the knowledge of the atomic structure of the individual domains, we have defined the full structure of p53 using homology modeling. Since we were analyzing the impact of deleterious mutations predicted in *TP53* on the p53–ERα interaction, we used our modelled structure instead of the PDB structure 2ocj (which contains only the DNA binding domain) used in our previous study [20]. The complete protein sequence of p53 was retrieved from Universal Protein Knowledgebase [23] (UniParc ID: P04637) and a BLAST search was done to predict the templates for individual domains of p53. Quality of the built models was assessed using PROCHECK available at Structural Analysis and Verification Server (SAVES: (http://nihserver.mbi.ucla.edu/SAVES/). The built model was subjected to minimization using NOMAD-Ref web server available at http://lorentz.immstr.pasteur.fr/nomad-ref.php with default settings [24].

### 3.2. Structure of ERα and Interaction of p53–ERα

Since the activation function-2 (AF-2) domain of ERα is necessary for interaction with p53 [15], three dimensional coordinates of the crystal structure of Human estrogen receptor alpha ligand-binding domain in complex with compound 11F (PDB code: 2IOG) with a high-resolution of 1.60 Å containing residues 306 to 554 (AF-2 domain) downloaded from the Protein Data Bank [25] was used for the p53–ERα interaction analysis. Modelled native and mutant p53 interactions with ERα were studied by subjecting them to protein–protein docking using ZDOCK 3.0.2: an automated server available at http://zdock.umassmed.edu/ [26]. Since residues in the regulatory domain of p53 are important for interaction with ERα, residues 363 to 393 were selected as binding site residues for ZDOCK protein–protein docking. All ten docking complexes were used for our analysis. Mutants (MTs) R110P, P151T and P278A were created by replacing the wild-type (WT) protein residue with its polymorphic residue using PyMOL [27] and minimized using NOMAD-Ref server. Properties of the native p53-ERα and mutant p53-ERα complexes were analyzed using Protein Interfaces, Surfaces and Assemblies service (PISA), available at European Bioinformatics Institute (http://www.ebi.ac.uk/msd-srv/prot_int/cgi-bin/piserver) [28].

### 3.3. Machine Learning Approach

Machine learning is a process of identifying the structure in a given data, in an automated or semi-automated way through a process called data mining. These machine learning approaches have the ability to generate models for prediction by extensively searching through the model and parameter space [29]. Previously, several studies have been done on protein–protein interactions using machine learning approaches [30,31,32]. Therefore, a diverse predictive model from machine learning or data mining has been employed here to perform predictions on the effect of these three mutations on the p53–ERα interaction. List of attributes mentioned in Table 2 given below was used for modeling using Weka 3.7.11. [33].

## 4. Discussion

Breast cancer is the most common and frequent cancer type for women around the world. Based on its complexity, heterogenicity and histological features, it has been classified into hormone-receptor-positive, human epidermal growth factor receptor-2 overexpressing (HER2+) and triple-negative breast cancer (TNBC) [34]. There are several genetic and nongenetic factors that confirm risk to breast cancer. In general, breast cancer susceptibility genes have been classified into high, moderate and low penetrance genes; each of them is interacting with several genes and environmental factors [35]. High penetrance genes include *BRCA1*, *BRCA2*, *PTEN*, *TP53*, *CDH1* and *STK11* whereas moderate penetrance genes include *CHEK2*, *BRIP1*, *ATM* and *PALB2* [36].

Among the high penetrance genes, p53 has a significant role in the malignancy of breast cancer with it mutations were more frequently observed in 30% of the breast carcinomas of which 26% are in luminal tumors (17% of luminal A, 41% of luminal B), 50% are in HER2 amplified tumors, 69% are in molecular apocrine breast carcinomas and 88% are in basal-like carcinomas [37]. In our previous study, we have screened the total number of non-synonymous coding single nucleotide polymorphisms (SNPs) in *TP53* gene and precited three deleterious coding non-synonymous SNPs rs11540654, rs17849781 and rs28934874 coding for mutations R110P, P278A, P151T in *TP53* with a phenotype in breast tumors using computational tools SIFT, Polyphen-2 and MutDB. Our results showed that these three mutations R110P, P151T and P278A have major consequences on the native p53 DNA-binding core domain RMSD, Rg, SASA, NH bond and number density in the presence and absence of Zn^2+^ ion [20]. Previous reports showed that R110P confers a loss of Tp53 protein function by decreased DNA binding and transactivation of Tp53 targets, and Caspase 3/7 activity in culture and an increased aggregation with Tp63, and Tp73 [38,39]. P278A mutation on *TP53* is known to show a loss of Tp53 transcription activity and a decrease in DNA binding and a failure to induce apoptosis in cell culture [39]. P151T on the other hand known to be found in the patients with early onset breast cancer [40,41].

In the present study, we aimed to investigate the impact of these three p53 mutations (R110P, P151T, P278A) on its interacting partners. Previous studies showed that p53 is known to interact with several other receptors thereby regulating a wide array of cellular processes leading to essential protection against cancer development [42,43,44]. Among the several interactions, one of the key interacting partner for p53 is ERα which directly bind to p53 thereby opposing p53-mediated apoptosis in breast cancer cells [15,45]. To this end, we have analyzed the impact of these three p53 mutations (R110P, P151T, P278A) on the estrogen receptor alpha–p53 interaction. The complete three-dimensional structure of p53 is still unavailable to date due to its intricate complexity and a comparatively little progress has been made through the years [46]. Therefore, in the present study, we have constructed the three-dimensional structure of p53 using a computational molecular modelling approach. The molecular modelling approach has been successful in several previous studies studying cancer mutations [47,48,49]. The complete sequence of human p53 was downloaded from the Universal Protein Knowledgebase database (UniParc ID: P04637) and searched for possible templates in the PDB structure database [50] using the BLAST search engine. Our results showed that four templates (Table 1) have a higher percentage of similarity with the p53 protein sequence. The built homology model was validated for a quality assessment using PROCHEK. Validated model was energy minimized and the mutants R110P, P151T, P278A were created by replacing the respective wild type p53 protein residues with its polymorphic residue using PyMOL software [27].

Human ERα is a 595 amino acids protein with an approximate molecular weight of 66–70 kDa [11]. Starting from NH2- to COO-terminus, ERα contains a typical structure of the nuclear receptor family with a highly variable N-terminal region (A/B domain), a highly conserved DNA-binding domain (C), a hinge domain (D), a ligand-binding domain (E) and a C-terminal domain (F). ERα is a ligand-inducible transcription factor which upon hormone binding, gets activated and regulates the transcription of target genes. Ligand-dependent and independent activation of ERα is done by the N-terminal A/B domain region (transactivation function-1 (AF-1)) whereas dimerization and binding to the coactivators and corepressors are done by the ligand-binding domain (transactivation function-2 (AF-2)) [51]. ERα is known to be expressed in approximately 70% of all the human breast cancers and a high level of ERα is associated with tumor differentiation thereby showing strong clinical evidence supporting its role in breast cancer [52,53]. ERα isoform primarily contributes to estrogen-induced growth stimulatory effects in breast cancer [54]. Point mutations in ERα may lead to hypersensitive estrogen breast hyperplasia [55] and several splice variants in ERα are known to be found in various tumor types of breast cancer. Among them, a variant in exon Δ3 of ERα (missing part of the central DNA binding domain) is known to function as a dominant-negative receptor, able to suppress estrogen-induced transcriptional activity [56]. PvuII polymorphism in the ERα, or another mutation in linkage disequilibrium with PvuII, in combination with high estradiol levels, is known to increase the breast cancer risk in postmenopausal women [57].

ERα plays an important role in the malignant progression of breast cancer. Two hypotheses were proposed to explain ERα association with breast cancer: (i) products of estrogen metabolism are genotoxic causing an increased risk of direct DNA damage; (ii) estrogen-induced activity of estrogen receptors stimulates proliferation leading to increased risk of DNA mutations due to high rates of DNA replication. Tamoxifen inhibits the ERα transcriptional activity in mammary cells and effectively reduces the risk of recurrence of invasive or in situ ERα positive breast cancer [52]. It is known to fail for treating breast cancer due to (i) the existence of ERβ (ii) an imbalance between estrogen biosynthesis and catabolism (iii) an altered bioavailability iv) altered cellular trafficking of ER (v) nongenomic effects of ER (vi) transcriptional dysregulation of ER target genes [58].

Among the different domains of ERα, the activation function-2 (AF-2) domain is known to be necessary for binding to the C-terminal regulatory domain of p53 [15]. The schematic structure of the respective interactions of ERα with p53 is shown in Figure 1. In general, protein–protein interfaces i.e., abbreviated as protein interfaces or interfaces are the regions where two proteins may show direct physical contact [59]. Since these interfaces are directly involved in protein–protein interactions the atoms and the residues at these interfaces play a prominent role in elucidating a protein interaction mechanism. Results from the protein–protein docking between p53 and ERα showed that interface atoms, interface residues, and interface SASA was increased in both p53 and ERα for R110P compared to the native complexes demonstrating that R110P mutation has more influence on the p53–ERα interaction interface compared to the other two mutants P151T and P278A.

Understanding the folding mechanisms of a protein involves the study of various structural parameters of proteins such as secondary structures, side chain interactions, H-bonds and surface residues. Generally, protein active sites are often situated at the surface of the protein, greater understanding into residue accessibility would be significant in understanding and predicting the structure/function relationships [60]. Results from our study demonstrated that the mutants P151T and P278A show a large deviation from the native p53-ERα complex in the surface atoms and surface residues compared to R110P. Overall, our results demonstrated that all three mutants showed a large deviation in the total solvent accessible surface area (Figure 3). Further, another parameter to understand and analyze the interactions across protein–protein interfaces is hydrogen bonds and of the salt bridges [61]. Results from the analysis of number of hydrogen bonding residues and the number of salt bridges showed that is an increase in the number of hydrogen bonding residues in R110P whereas there is an increase in number salt bridge forming residues in the mutant P278A (Figure 4) indicating there is a change in global conformation induced by these residues at the p53-ERα interface. Overall, our results demonstrate that all these three mutations have an impact on the p53–ERα interaction.

Machine learning is an emerging scientific discipline focusing on intersecting statistics in relation to data and computer science with its emphasis on implementing efficient computing algorithms [62]. Previous studies showed that machine learning models have been successful in breast cancer studies [63,64,65]. In the present study, to check whether the structural features we have analyzed are important for predicting the impact of these three mutants on the p53–ERα interaction, we used Weka a data mining toolkit that implements the machine learning algorithms [66]. We have used the data generated from the protein–protein docking results of the native, R110P, P151T and P278A p53-ERα complexes to building an artificial neural network using multilayer perceptron function of Weka software (Figure 6) (Table 2 and Table 3). Overall our results demonstrate that the structural features of interface atoms, surface atoms, interface residues, surface residues, interface SASA, total SASA, isolated structure Solvent energy, gain on complex formation, average gain in complex formation, number of hydrogen bonding residues and number of salt bridge residues are significant for predicting the impact of the mutations R110P, P278A and P151T induced by the deleterious breast cancer SNPs rs11540654, rs17849781 and rs28934874 predicted in our previous study [20] on the p53–ERα interaction.

## 5. Conclusions

Almost 70% of human breast cancers are ER-positive and hormone-dependent. Hormonal therapy to treat ER-positive breast cancer is one of the most extensively described uses of personalized medicine and has been studied for over a century. Results from the present study conclusively show that the three mutants R110P, P151T and P278A that are predicted to be deleterious on the DNA binding domain of p53 have an impact on the interaction with the ligand binding domain of human estrogen receptor alpha. The parameters interface and surface residues, interface and total SASA contribute to the overall impact of these three mutants on the p53–ERα interaction. Overall, results from our study can be useful as a tool to predict the impact of mutations in the ER-positive breast cancer patients and can be useful for decision making.

## Figures and Tables

**Table 1:** List of templates used for modelling p53 structure.

Domain Name	PDB ID: Chain Name
Transactivation domain	2LY4:B, 2GS0:B
DNA binding domain	2OCJ:A,1TSR:A
Tetramerization domain	1OLG:A, 1C26:A
Regulatory domain	1DT7:X, 2H4J:D, 1JSP:A

**Table 2:** Attributes used for building the neural network for p53–ERα interaction.

Attribute	Variable Type	Value Range
Interface atoms ^(p53)^	Numerical (continuous)	(74, 179)
Interface atoms ^(ER)^	Numerical (continuous)	(75, 173)
Surface atoms ^(p53)^	Numerical (continuous)	(1933, 1999)
Surface atoms ^(ER)^	Numerical (continuous)	(1154, 1173)
Interface residues ^(p53)^	Numerical (continuous)	(16, 53)
Interface residues ^(ER)^	Numerical (continuous)	(20, 44)
Surface residues ^(p53)^	Numerical (continuous)	(365, 374)
Surface residues ^(ER)^	Numerical (continuous)	(220, 224)
Interface SASA ^(p53)^	Numerical (continuous)	(693.5, 1575.2)
Interface SASA ^(ER)^	Numerical (continuous)	(694.2, 1521.7)
Total SASA ^(p53)^	Numerical (continuous)	(23,820.5, 24,850.6)
Total SASA ^(ER)^	Numerical (continuous)	(12,983.1, 13,016.6)
Isolated structure Solvent energy ^(p53)^	Numerical (continuous)	(−216.9, −200.6)
Isolated structure Solvent energy ^(ER)^	Numerical (continuous)	(−255.9, 254.6)
Gain on Complex formation ^(p53)^	Numerical (continuous)	(−10.2, −2.0)
Gain on Complex formation ^(ER)^	Numerical (continuous)	(−10.2, 0.1)
Average gain in Complex formation ^(p53)^	Numerical (continuous)	(−13.7, −5.9)
Average gain in Complex formation ^(ER)^	Numerical (continuous)	(−7.6, −3.3)
Number of Hydrogen bonding residues ^(p53)^	Numerical (continuous)	(0, 13)
Number of Hydrogen bonding residues ^(ER)^	Numerical (continuous)	(0, 12)
Number of Salt bridge residues ^(p53)^	Numerical (continuous)	(0, 5)
Number of Salt bridge residues ^(ER)^	Numerical (continuous)	(0, 5)
Complex	Categorical (quaternary)	(Native, R110P, P151T, P278A)

(p53) represents tumor suppressor p53; (ER) represents ERα attribute combinations.

**Table 3:** Dataset pre-analysis used for building the neural network for p53–ERα interaction.

Attribute	Sub Groups & Ranges	Value Range
Total SASA ^(p53)^	(Very Low) = 23,820.5 (Low) = < 24,335.55 (Medium) = ≥ 24,335.55 (High) = 24,850.6	(23,820.5, 24,850.6)
Total SASA ^(ER)^	(Very Low) = 12,983.1 (Low) = < 12,999.85 (Medium) = ≥ 12,999.85 (High) = 13,016.6	(12,983.1, 13,016.6)
Average gain in Complex formation ^(p53)^	(Very Low) = −13.7 (Low) = < −9.8 (Medium) = ≥ −9.8 (High) = −5.9	(−13.7, −5.9)
Average gain in Complex formation ^(ER)^	(Very Low) = −7.6 (Low) = < −5.45 (Medium) = ≥ −5.45 (High) = −3.3	(−7.6, −3.3)
Number of Hydrogen bonding residues ^(p53)^	(None) = 0 (Low) = < 6 (Medium) = ≥ 6 (High) = 13	(0, 13)
Number of Hydrogen bonding residues ^(ER)^	(None) = 0 (Low) = < 6 (Medium) = ≥ 6 (High) = 12	(0, 12)
Number of Salt bridge residues ^(p53)^	(None) = 0 (Low) = < 2 (Medium) = ≥ 2 (High) = 5	(0, 5)
Number of Salt bridge residues ^(ER)^	(None) = 0 (Low) = < 2 (Medium) = ≥ 2 (High) = 5	(0, 5)

(p53) represents tumor suppressor p53; (ER) represents ERα attribute combinations.
